# PSYCHOSOCIAL AND CULTURAL PAIN EXPERIENCES AND SELF-MANAGEMENT ACROSS DIVERSE OLDER ADULT POPULATIONS

**DOI:** 10.1093/geroni/igz038.271

**Published:** 2019-11-08

**Authors:** Staja Booker, Keesha Roach

**Affiliations:** The University of Florida, Gainesville, Florida, United States

## Abstract

Racial/ethnic minority older adults experience a disproportionate burden of functionally-disabling chronic pain. However, minimal research in pain and aging has fully explicated the unique and endemic psychosocial and cultural factors that strongly influence appraisal, communication, and management/coping of pain. Yet, to fully engage with and care for diverse racial/ethnic older adults, intentional responsiveness to these factors is necessary. This symposium features under-represented racial/ethnic older adult populations and multiple methodologies, including advanced imaging techniques, to understand various psychosocial and cultural factors associated with chronic pain. Our first presenter, Dr. Lor, uses qualitative inquiry to examine pain-associated language and expression of pain in Hmong older adults, which is often laden with stress and misunderstanding. Following is Dr. Taylor who will discuss the mediating effect of stress and coping on bodily pain in inner-city Black older adults. Dr. Terry will present novel findings on the association between catastrophizing (i.e., negative cognitive and emotional response to actual or anticipated pain resulting in feelings of helplessness) and brain structure in non-Hispanic Black and White adults with or at high-risk for knee osteoarthritis pain. This presentation will segway into culturally-relevant pain self-management practices and the role of social support specifically for Blacks from urban Detroit, as presented by Dr. Janevic. We will conclude with Dr. Booker presenting mixed-level data on the lack of familial and social networks and provider support for osteoarthritis pain self-management in Southern-dwelling older Blacks. This symposium extends the knowledge on the nuanced complexity of biopsychosocial and cultural dynamics underlying the pain experience.

